# DANGER is involved in high glucose-induced radioresistance through inhibiting DAPK-mediated anoikis in non-small cell lung cancer

**DOI:** 10.18632/oncotarget.6887

**Published:** 2016-01-12

**Authors:** TaeWoo Kwon, HyeSook Youn, Beomseok Son, Daehoon Kim, Ki Moon Seong, Sungkyun Park, Wanyeon Kim, BuHyun Youn

**Affiliations:** ^1^ Department of Integrated Biological Science, Pusan National University, Busan, 609-735, Republic of Korea; ^2^ Department of Biological Sciences, Pusan National University, Busan, 609-735, Republic of Korea; ^3^ Nuclear Science Research Institute, Pusan National University, Busan, 609-735, Republic of Korea; ^4^ National Radiation Emergency Medical Center, Korea Institute of Radiological & Medical Sciences, Seoul, 139-706, Republic of Korea; ^5^ Department of Physics, Pusan National University, Busan, 609-735, Republic of Korea

**Keywords:** radioresistance, high glucose, DANGER, DAPK, anoikis

## Abstract

^18^F-labeled fluorodeoxyglucose (FDG) uptake during FDG positron emission tomography seems to reflect increased radioresistance. However, the exact molecular mechanism underlying high glucose (HG)-induced radioresistance is unclear. In the current study, we showed that ionizing radiation-induced activation of the MEK-ERK-DAPK-p53 signaling axis is required for anoikis (anchorage-dependent apoptosis) of non-small cell lung cancer (NSCLC) cells in normal glucose media. Phosphorylation of DAPK at Ser734 by ERK was essential for p53 transcriptional activity and radiosensitization. In HG media, overexpressed DANGER directly bound to the death domain of DAPK, thus inhibiting the catalytic activity of DAPK. In addition, inhibition of the DAPK-p53 signaling axis by DANGER promoted anoikis-resistance and epithelial-mesenchymal transition (EMT), resulting in radioresistance of HG-treated NSCLC cells. Notably, knockdown of DANGER enhanced anoikis, EMT inhibition, and radiosensitization in a mouse xenograft model of lung cancer. Taken together, our findings offered evidence that overexpression of DANGER and the subsequent inhibitory effect on DAPK kinase activity are critical responses that account for HG-induced radioresistance of NSCLC.

## INTRODUCTION

Radiotherapy can be a preferential management strategy for patients with inoperable cancer, including advanced stages of non-small cell lung cancer (NSCLC), in the absence of more effective targeted therapies [1]. Nevertheless, therapeutic outcomes are not fully satisfactory due to the emergence of radioresistance that is a critical obstacle which causes failure of radiotherapy and increased mortality of patients with NSCLC [2]. So far, a large number of studies have been conducted to find a way to control radioresistance and develop potent adjuvants that enhance radiotherapy efficacy [3, 4]. A profound understanding of molecular events associated with therapeutic resistance would greatly advance the discovery of drugs that suppress radioresistance signaling to improve NSCLC prognosis [5, 6].

Since ^18^F-labeled fluorodeoxyglucose (FDG) was first synthesized in 1978, FDG positron emission tomography (FDG-PET) has been one of the most useful oncological imaging modalities [7]. Due to the fact that glucose uptake and consumption of malignant tumors is elevated, FDG-PET is based on a close glucose chemical analogy and produces valuable data for detecting malignancies, searching for targets, and observing responses [8]. Although the use of FDG can provide information about prognosis associated with radiation therapy, high glucose (HG) uptake, which correlates with high FDG uptake, may increase radioresistance [9]. Moreover, GLUT1 which is the major glucose transporter is highly expressed in NSCLC. FDG uptake and the GLUT1 expression level correlated with the tumor size [10]. Several clinical meta-analyses suggest that high FDG uptake in a tumor is associated with increased local failure for many tumor sites and reduced patient survival rates [11, 12]. FDG-avid regions of a tumor are accordingly considered as a potential target for the application of radiation-dose escalation to counteract radioresistance [13]. A recent meta-analysis study showed that FDG-avid tumors in patients with head and neck carcinomas require a radiation dose increased 20% to match the local response rate of non-FDG avid tumors [14].

Glycolysis in tumor cells exposed to hypoxic environments is escalated because cells produce ATP through glycolysis, a process that does not need oxygen. However, tumor cells exhibit a higher rate of glycolysis than normal cells even under normoxic conditions; this is known as the “Warburg effect” [15]. Genetic or epigenetic alterations are thought to alter glycolysis levels in a manner that drives tumor malignancy [16]. As energy production of glycolysis is far less efficient than that of oxidative phosphorylation in mitochondria, a set of genes that govern glucose uptake and utilization are frequently overexpressed in cancer cells. Although several studies have shown a relationship between HG uptake and characteristics of tumor cells, the mechanism underlying cancer cell resistance to cell death is still unclear.

DANGER (also known as ITPRIP, inositol 1,4,5-trisphosphate receptor (IP_3_R) interacting protein) is identified to bind to IP_3_R and contains a partial MAB-21 domain [17]. After binding to IP_3_R, DANGER accelerates the calcium-inhibitory function of the IP_3_R channel, leading to regulation of the neuronal process. A previous biochemical study demonstrated that DANGER physically associates with death-associated protein kinase (DAPK) and impedes the catalytic activity of DAPK [18]. Mouse embryonic fibroblasts and neurons deficient in DANGER have elevated DAPK activity and increased responses to cell death signals compared to control cells and DANGER-deficient mice are more sensitive to brain damage after neuronal injury [18]. In addition, a previous RNA-sequencing analysis showed that *ITPRIP* expression is up-regulated in monocytes treated with high levels of glucose [19].

DAPK is a Ser/Thr protein kinase that was originally characterized as a tumor suppressor owing to its ability of promoting cell death [20]. DAPK is up-regulated in response to various signals such as those associated with interferon-γ, TGF-β, TNF-α, and Fas [21]. In the gut, TNF-α promotes DAPK-induced apoptosis in tumor cells, whereas normal intestinal epithelial cells are resistant to TNF-α, but are subject to remarkable DAPK-induced inflammation [22, 23]. However, little is known about its effects on ionizing radiation (IR)-induced cell death. Multi-domain structure of DAPK includes a catalytic domain, a Ca^2+^/calmodulin-binding region, eight ankyrin repeats, two putative nucleotide-binding domains (P-loops), a cytoskeleton/Ras of complex proteins (ROC) domain, and a C-terminal death domain (DD). This structure is responsible not only for direct protein phosphorylation of DAPK substrates but also stabilization of multi-protein complexes in a cell [24]. A cluster of DAPK interaction partners includes proteins that act upstream of DAPK and affect its kinase activity, stability, or subcellular localization; this includes proteins that function as DAPK downstream effectors [25]. Interaction of ERK with the DD of DAPK enhances the ability of DAPK to promote apoptosis [26]. ERK binds a canonical docking sequence within the DD of DAPK, and phosphorylates DAPK on Ser734 within the ROC domain. This modification enhances the catalytic activity of DAPK towards its substrate, myosin regulatory light chain (MLC). This is reflected by a lower *K_m_* value, while *K_cat_* and *V_max_* remain unchanged, suggesting that Ser734 modification may stimulate substrate binding [26]. The mechanism by which this occurs is unclear.

The purpose of this study was to elucidate the mechanisms and key molecules that confer HG-induced radioresistance in NSCLC cells. We demonstrated that HG-induced overexpressed DANGER bound to the DD of DAPK and subsequently inhibited ERK/DAPK-induced death of NSCLC cells. Our findings provide a possible explanation of how FDG uptake increases radioresistance in NSCLC cells. Furthermore, we suggest that DANGER and DAPK could be attractive pharmaceutical targets for overcoming HG-induced radioresistance of NSCLC and ultimately contribute to the effective treatment of lung cancer with radiation.

## RESULTS

### HG induces DANGER overexpression in NSCLC cells

**Figure 1:**
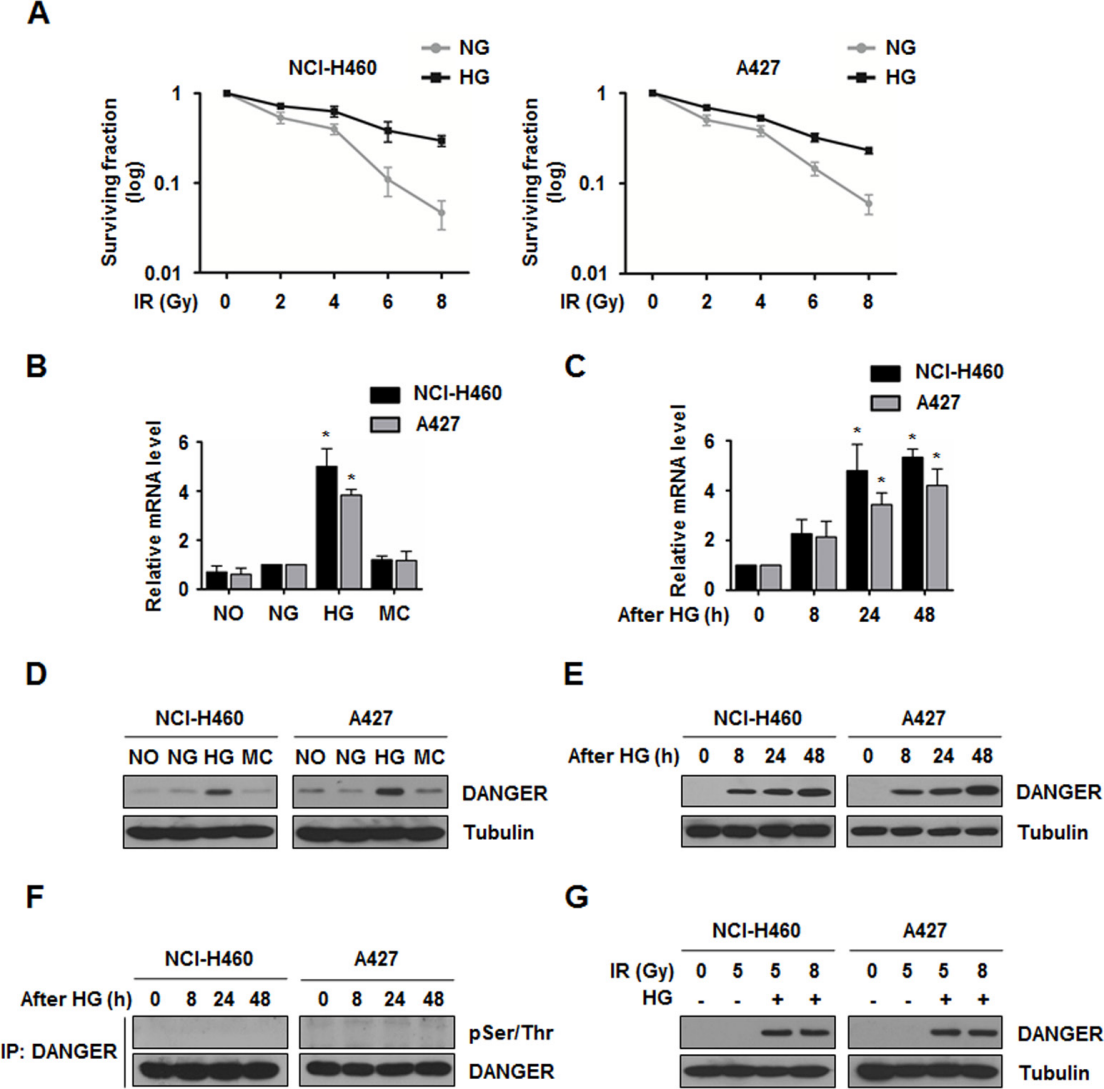
HG induces DANGER overexpression in NSCLC cells **A.** Survival curves for NG- or HG-treated NCI-H460 and A427 cells exposed to IR were assessed with a colony forming assay. Data are represented as mean ± SEM (*n* = 3). **B.** Expression of *ITPRIP* in HG-treated NCI-H460 and A427 cells was analyzed by qRT-PCR. After treatment with specific media (NO, no glucose; NG, normal glucose; HG, high glucose; or MC, mannitol control) for 24 h, relative mRNA levels of DANGER were monitored. Data are represented as mean ± SEM (*n* = 3); *p < 0.05 compared to the NG-treated cells. **C.** Time-dependent expression of *ITPRIP* in HG-treated NCI-H460 and A427 cells was analyzed by qRT-PCR. Data are represented as mean ± SEM (*n* = 3); *p < 0.05 compared with cells after 0 h of HG treatment. **D.** and **E.** Expression of DANGER and time-dependent changes in HG-treated NCI-H460 and A427 cells were analyzed by Western blotting. **F.** HG-induced phosphorylation of DANGER was evaluated with an *in vivo* kinase assay. After HG treatment for the indicated time, the cells were harvested and cell lysates were subjected to an IP assay with an anti-DANGER antibody followed by Western blotting for pSer/Thr. **G.** Effect of IR on HG-induced overexpression of DANGER was analyzed by Western blotting.

To confirm HG-induced radioresistance in NSCLC cells, NCI-H460 and A427 cells were used because these cell lines have relatively high levels of radiosensitivity [4, 27]. We first cultured NCI-H460 and A427 cells in medium containing different concentrations of glucose and measured radiosensitivity using a colony forming assay. As shown in Figure 1A, NCI-H460 and A427 cells cultured with 30 mM glucose showed higher resistance to a pro-apoptotic dose of radiation (5 Gy) than ones grown in normal glucose (NG) medium (5.5 mM glucose). The 30 mM of glucose was used as HG, since previous studies investigating metabolic disorders with abnormal glucose metabolism commonly applied 30 mM of glucose for high concentration of glucose to cellular systems [28, 29]. Colony formation of HG-treated cells was greater by approximately 6-fold for NCI-H460 cells and 4-fold for A427 cells compared to NG-treated cells. These findings led us to confirm that HG uptake might be associated with radioresistance in NSCLC cells. We next investigated key factor(s) associated with HG-induced radioresistance of NSCLC cells. A previous transcriptome analysis showed that DANGER expression is up-regulated in HG-treated monocytes [19]. Based on the information, we measured the expression of DANGER in HG-treated NCI-H460 and A427 cells. HG treatment dramatically induced mRNA and protein expression of DANGER in both cell lines in a time-dependent manner (Figure 1B-1E). HG-induced increase of DANGER mRNA and protein levels were verified in additional four NSCLC cell lines, NCI-H157, NCI-H23, NCI-H1299, and NCI-H358 (Supplementary Figure S1A, S1B) and we confirmed that elevated DANGER protein expression was sustained for at least 48 h after HG treatment (Supplementary Figure S1C). Next, molecular modification of DANGER by HG treatment was examined because DANGER was known to be phosphorylated at Ser547 [30]. However, HG-induced DANGER phosphorylation at Ser residues was not detected in either NSCLC cell line (Figure 1F). To determine whether HG can change the expression of DANGER in the presence of IR, the protein levels of DANGER were measured in HG-treated and irradiated NCI-H460 or A427 cells. As shown in Figure 1G, the levels of DANGER were increased in the HG-treated NSCLC cells. Elevated DANGER expression was not observed with IR in either cell line. Collectively, these data suggest that HG induces the overexpression of DANGER in NSCLC cells.

### Down-regulation of DANGER expression reduces HG-induced radioresistance in NSCLC cells

**Figure 2:**
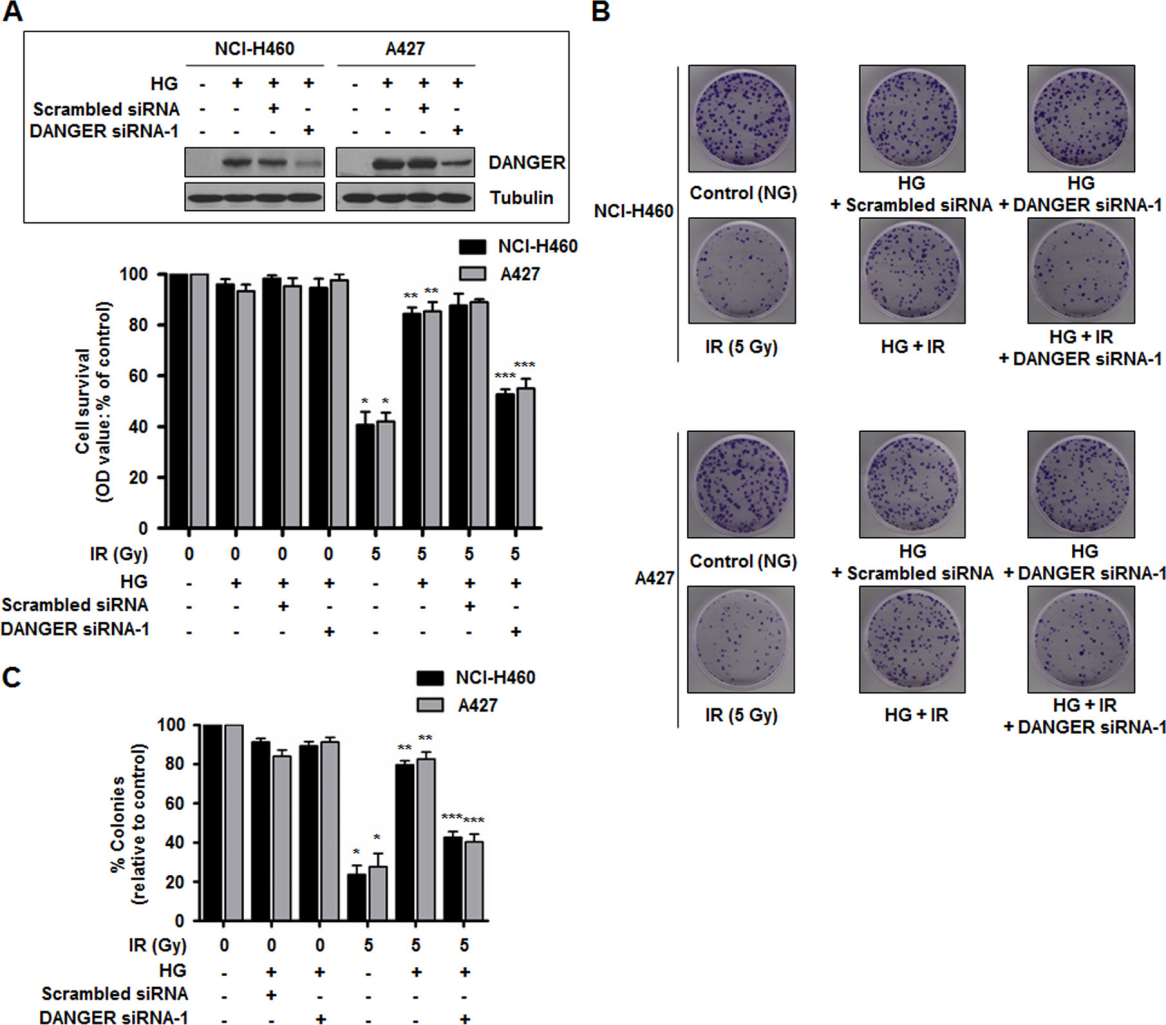
Down-regulation of DANGER expression reduces HG-induced radioresistance in NSCLC cells **A.** Short-term effects of DANGER knockdown on cell growth in both NCI-H460 and A427 cells following IR exposure were assessed with an MTT assay. Data are represented as mean ± SEM (*n* = 3); *p < 0.05 compared to non-irradiated cells, **p < 0.05 compared to cells treated with irradiation-alone, ***p < 0.05 compared to HG-treated and irradiated cells. Inset: SiRNA knockdown efficiency of DANGER in HG-treated NCI-H460 and A427 cells was analyzed by Western blotting. **B.** Long-term effects of DANGER knockdown on cell growth in both NCI-H460 and A427 cells following IR exposure were assessed with a colony forming assay. **C.** Quantitative analysis of the number of NCI-H460 and A427 cell clones after DANGER knockdown with or without HG treatment was performed with Image J. Data are represented as mean ± SEM (*n* = 3); *p < 0.05 compared to non-irradiated cells, **p < 0.05 compared to cells treated with irradiation-alone, ***p < 0.05 compared to HG-treated and irradiated cells.

To determine whether DANGER is responsible for HG-induced radioresistance in NSCLC cells, two DANGER siRNAs were used to suppress DANGER expression. The short-term effect of DANGER knockdown on irradiated cell growth was evaluated with an MTT assay in NCI-H460 and A427 cells. Knockdown of DANGER expression had no significant effect on cell proliferation with HG-alone (Figure 2A and Supplementary Figure S2A). However, DANGER knockdown significantly reduced HG-induced growth of irradiated NSCLC cells, suggesting that DANGER protects cancer cell from IR-induced cell death. To evaluate the long-term effect of DANGER knockdown on cell proliferation, a colony formation assay was conducted. The formation of colonies by both cell lines after DANGER knockdown was slightly reduced in the presence of HG-alone. In contrast, the HG-induced increase of colony formation in the presence of IR was greatly diminished by DANGER knockdown, which was reversed by overexpressed DANGER (Figure 2B, 2C, Supplementary Figure S2B, and S2C). These results suggest that DANGER knockdown reduces HG-induced radioresistance of NSCLC cells.

### HG-induced DANGER physically interacts with DAPK in NSCLC cells

**Figure 3:**
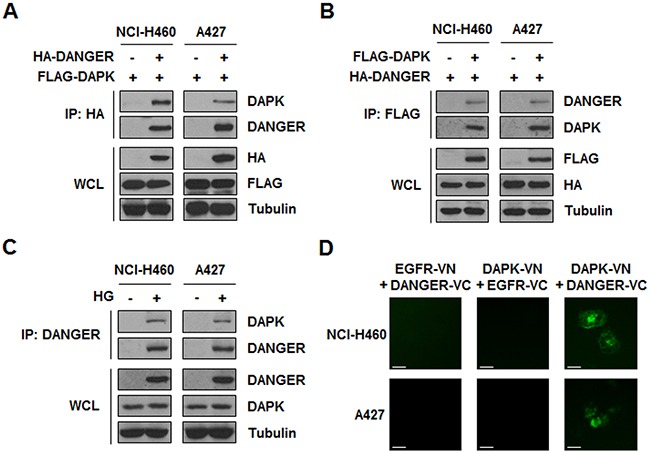
HG-induced DANGER physically interacts with DAPK in NSCLC cells **A.** and **B.** Binding of DANGER and DAPK was measured by a reciprocal IP assay. **C.** HG-induced overexpression of DANGER and subsequent interaction of endogenous DANGER with endogenous DAPK was detected using an IP assay. **D.** A BiFC assay was performed to evaluate the interaction of DAPK-DANGER in live cells. Cells were transiently transfected with pBiFC-DAPK-VN, pBiFC-DANGER-VC, pBiFC-EGFR-VN, and/or pBiFC-EGFR-VC. Fluorescence indicative of DANGER-DAPK binding was measured in NCI-H460 or A427 cells. Scale bars, 10 μm.

Since we found that DANGER was overexpressed with HG treatment and involved in radioresistance of NSCLC cells, we attempted to identify a DANGER-interacting proteome in HG-treated and irradiated NSCLC cells to elucidate the molecular functions of DANGER. Based on the information from a published literature of physical and functional interactions, we focused on DAPK, a key regulator of stress-induced apoptosis [25]. Importantly, a previous functional study demonstrated that DANGER directly binds to DAPK and inhibits the kinase activity of DAPK, consequently increasing neuron viability [18]. We subsequently assessed the binding of DANGER and DAPK in NSCLC cells by a reciprocal immunoprecipitation (IP) assay (Figure 3A, 3B). HG-induced overexpression of DANGER and subsequent binding of endogenous DANGER to endogenous DAPK was detected in NSCLC cells (Figure 3C). In addition, we confirmed the subcellular binding of DANGER and DAPK in live cells by a bimolecular fluorescence complementation (BiFC) assay (Figure 3D). The immunostaining results of DAPK and DANGER in NSCLC cells also suggested co-localization of the two proteins (Supplementary Figure S3). Taken together, these results indicated that DANGER physically associates with DAPK in HG-treated NSCLC cells.

### DANGER inhibits the catalytic activity of DAPK in NSCLC cells

**Figure 4:**
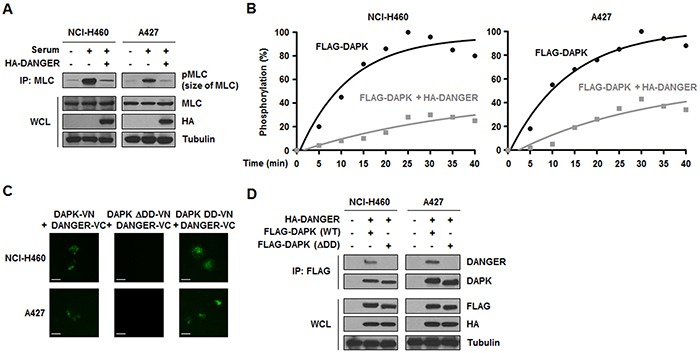
DANGER inhibits the catalytic activity of DAPK in NSCLC cells **A.** Effects of DANGER overexpression on DAPK activity were measured with a kinase assay. **B.** Effects of DANGER on kinetics of MLC phosphorylation by DAPK were measured with a kinetic analysis. The extent of phosphorylation was quantified and expressed as a percentage with 100% representing the maximum phosphorylation of MLC. **C.** A BiFC assay was performed to determine the interaction of DAPK with DANGER in live cells. NCI-H460 or A427 cells were transiently transfected with pBiFC-DAPK (WT, DD-only, or ΔDD)-VN and/or pBiFC-DANGER-VC. Fluorescence indicative of DANGER-DAPK binding was measured in the cells. Scale bars, 10 μm. **D.** Involvement of the DAPK DD in binding to DANGER was confirmed with an IP assay.

Given the physical interaction between DANGER and DAPK, we next focused on the functional relationship of these proteins. A previous study revealed that the catalytic activity of DAPK is increased in response to serum stimulation [31]. A DAPK kinase assay was conducted by measuring the phosphorylation of MLC, a DAPK substrate, after transfection of HA-DANGER followed by serum stimulation. We observed that DANGER overexpression led to decreased MLC phosphorylation, indicating that DANGER inhibited serum-induced DAPK activation (Figure 4A). Analysis of the kinetics indicated that MLC phosphorylation by DAPK was increased in a time-dependent manner and this activity was significantly reduced by DANGER overexpression in NSCLC cells (Figure 4B). Since many proteins interact with the DD of DAPK [25], we determined the function of the DAPK DD in the context of an association between DANGER and DAPK using a BiFC assay. Fluorescence was observed in cells expressing DAPK (WT or DD-only) and DANGER, but not in those that expressed DAPK-ΔDD and DANGER (Figure 4C). This result was confirmed by a binding assay (Figure 4D). Taken together, these findings indicated that DANGER inhibits the catalytic activity of DAPK through a direct interaction with the DD of DAPK in NSCLC cells.

### DANGER inhibits DAPK-induced anoikis in irradiated NSCLC cells

**Figure 5:**
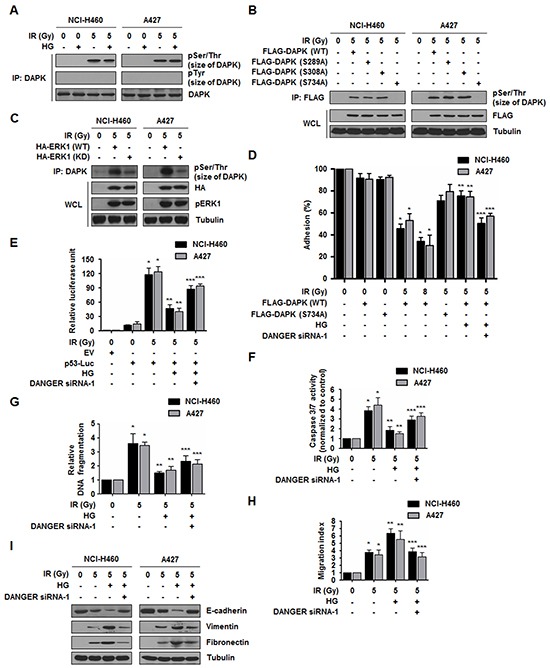
DANGER reduces DAPK-dependent anoikis in irradiated NSCLC cells **A.** HG- and IR-dependent phosphorylation of DAPK was assessed. **B.** IR-induced DAPK phosphorylation of Ser residues was confirmed using DAPK mutants (S289A, S308A, or S734A). **C.** Phosphorylation of DAPK by ERK1 was measured using an ERK1-KD (K71R) mutant. pERK1 indicates phosphorylated ERK1. **D.** Effects of DANGER knockdown on the anti-adhesion effect of DAPK were measured with an adhesion analysis. The cells were irradiated and cell adhesion on fibrinogen was measured. Data are represented as mean ± SEM (*n* = 3); *p < 0.05 compared to non-irradiated cells transfected with DAPK-WT, **p < 0.05 compared to irradiated cells transfected with DAPK-WT, ***p < 0.05 compared to HG-treated and irradiated cells transfected with DAPK-WT. **E.** Effects of DANGER knockdown on HG- and IR-induced p53 transcriptional activation were measured with a luciferase assay. Data are represented as mean ± SEM (*n* = 3); *p < 0.05 compared to non-irradiated cells, **p < 0.05 compared to cells treated with irradiation-alone, ***p < 0.05 compared to HG-treated and irradiated cells. **F.** Effects of DANGER knockdown on HG- and IR-induced Caspase 3/7 activation in NSCLC cells were measured with a Caspase 3/7 activity assay. Data are represented as mean ± SEM (*n* = 3); *p < 0.05 compared to non-irradiated cells, **p < 0.05 compared to cells treated with irradiation-alone, ***p < 0.05 compared to HG-treated and irradiated cells. **G.** Functional involvement of DANGER knockdown in HG- and IR-induced DNA damage responses was measured with a DNA fragmentation assay. Data are represented as mean ± SEM (*n* = 3); *p < 0.05 compared to non-irradiated cells, **p < 0.05 compared to cells treated with irradiation alone, ***p < 0.05 compared to HG-treated and irradiated cells. **H.** The inhibitory effects of DANGER knockdown on HG- and IR-induced NSCLC cell migration were measured using a Transwell migration assay. Data are represented as mean ± SEM (*n* = 3); *p < 0.05 compared to non-irradiated cells, **p < 0.05 compared to cells treated with irradiation-alone, ***p < 0.05 compared to HG-treated and irradiated cells. **I.** Effects of DANGER knockdown on the protein expression of E-cadherin, Vimentin, and Fibronectin in HG-treated NSCLC cells were analyzed by Western blotting.

We further set out to identify the physiological relevance of DANGER-DAPK interactions since we found that DANGER inhibited the catalytic activity of DAPK through direct interaction in HG-treated and irradiated NSCLC cells. First, we evaluated HG- and IR-induced biochemical modification of DAPK based on information about DAPK-mediated signaling dependent on its phosphorylation [26]. In both cell lines, IR-induced DAPK phosphorylation on Ser/Thr sites was observed, whereas HG-dependent phosphorylation of Ser/Thr and Tyr residues was not detected (Figure 5A). Based on data from the literature, we used different DAPK mutants (S289A, S308A, or S734A) as a substrate for a kinase assay to further determine which residues are the targets of phosphorylation by IR [26, 32, 33]. Phosphorylation was completely absent in the DAPK-S734A mutant (Figure 5B). These findings indicated that Ser734 of DAPK is required for phosphorylation in irradiated NCI-H460 or A427 cells. It has been reported that ERK phosphorylates DAPK at Ser734, which consequently promotes cytoplasmic retention of ERK and DAPK-induced apoptosis [26]. In this study, we observed that DAPK was phosphorylated at Ser sites by ERK1-WT but not ERK1-kinase-dead (KD) after irradiation (Figure 5C). Direct phosphorylation of DAPK by ERK was confirmed by treating NCI-H460 and A427 cells with an ERK-specific inhibitor, PD98059, and a MEK inhibitor, GDC-0973. IR-induced DAPK phosphorylation by ERK was significantly diminished following treatment with the two inhibitors (Supplementary Figure S4A). Moreover, DAPK phosphorylation was not essential for interaction with DANGER as shown in Supplementary Figure S4B. Taken together, these results indicated that DAPK is phosphorylated at Ser734 by ERK in irradiated NSCLC cells.

Notably, the phosphorylation of DAPK at Ser734 by ERK has been reported to be essential for the pro-anoikis function of DAPK [26]. Based on a previous study demonstrating that DAPK inhibits cell adhesion and induction of anoikis [34], we assessed the effect of DANGER on the adhesion-inhibitory activity of DAPK. As shown in Figure 5D and Supplementary Figure S4C, activation of ERK by IR further potentiated this anti-adhesion effect in a dose-dependent manner in cells expressing DAPK-WT but not DAPK-S734A. However, the diminished anti-adhesion function was restored by DANGER overexpression with HG treatment; this effect was offset by DANGER knockdown. Since it has been reported that p53 induction and apoptosis is stimulated by the adhesion-inhibitory activity of DAPK [35], we next evaluated the effect of DANGER on DAPK-induced p53 activation and apoptosis. We observed that DANGER reduced DAPK-mediated transcriptional activation of the p53-promoter reporter constructs (Figure 5E and Supplementary Figure S4D) and expression of p21 which is a known target of p53 activity (Supplementary Figure S4E). Additionally, IR-induced pro-apoptotic activity of DAPK was markedly decreased by HG treatment. However, depleting DANGER expression significantly increased radiosensitivity of irradiated NSCLC cells (Figure 5F, 5G, Supplementary Figure S5A, and S5B). These results indicate that DANGER reduced DAPK-induced anoikis in irradiated NSCLC cells.

Several lines of evidence have demonstrated that epithelial-mesenchymal transition (EMT) status including loss of cell-cell contact increases scattering migration/invasion and resistance to anoikis [36, 37]. Having identified the effect of DANGER on IR-induced anoikis, we next measured cell migration capacity and EMT marker expression. Reduced motility in NSCLC cells was shown by treatment of DANGER siRNAs (Figure 5H and Supplementary Figure S6A). HG treatment promoted IR-induced EMT by decreasing the expression of E-cadherin (an epithelial marker) while increasing the expression of Vimentin and Fibronectin (mesenchymal markers) (Figure 5I and Supplementary Figure S6B). Reduced EMT was also observed in NSCLC cells in which DANGER expression was knocked-down. Taken together, these results suggest that the inhibition of DANGER expression suppresses HG-activated EMT in NSCLC cells.

### DANGER knockdown enhances *in vivo* radiosensitization and decreases *in vivo* EMT in a xenograft mouse model

**Figure 6:**
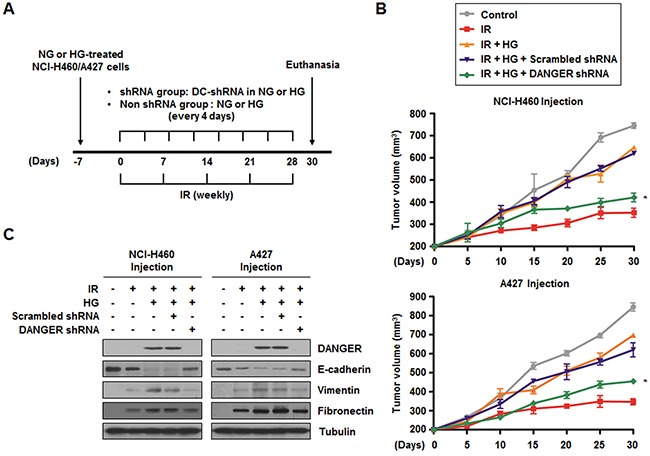
Knockdown of DANGER enhances *in vivo* radiosensitization and decreases *in vivo* EMT in a xenograft mouse model **A.** The experimental protocol for determining whether DANGER knockdown increases *in vivo* radiosensitization and EMT in a xenograft mouse model (control or DANGER-specific shRNA encapsulated into DOTAP-cholesterol, DC-shRNA).**B.** The effects of DANGER knockdown by DANGER shRNA-1 on *in vivo* radiosensitization were measured in a xenograft mouse model. Data are represented as mean ± SEM (*n* = 3 with three animals/group); *p < 0.05 compared to tumor volume on day-30 in mice treated with radiation, HG, and Scrambled shRNA. **C.** The *in vivo* effects of DANGER knockdown by DANGER shRNA-1 on the expression of DANGER and EMT-related proteins were evaluated by Western blot analysis.

In our cell death assays, we observed that DANGER reduced DAPK-induced anoikis in irradiated NSCLC cells (Figure 5, Supplementary Figures S4, S5, and S6). To evaluate the combined effects of DANGER and IR on tumor growth *in vivo*, a xenograft mouse model was established (Figure 6A). The data from nude mice bearing tumors formed by NCI-H460 and A427 cells indicated that HG induced radioresistance *in vivo* (Figure 6B). Tumor volumes of the mice treated with HG were significantly increased by approximately 76.2% (the group injected with NCI-H460 cells) or 78.8% (the group injected with A427 cells) on day-30 compared to mice receiving radiation-alone. We also confirmed that DANGER overexpression by lentiviral transduction in NSCLC cells made them more radioresistant (Supplementary Figure S7A). Compared to mice receiving radiation-alone, tumor volumes of the mice injected with DANGER-overexpressed NSCLC cells were significantly elevated by 97.4% (NCI-H460 cells) or 103% (A427 cells). Increased radioresistance due to HG treatment was offset by DANGER knockdown. Moreover, the expression of DANGER and EMT-related mesenchymal marker proteins was significantly elevated in the extracted tumor tissue lysates when HG-treated or DANGER-overexpressed cells were administered to the mice (Figure 6C and Supplementary Figure S7B). Thus, we suggest that DANGER knockdown significantly increased *in vivo* radiosensitization while inhibiting the EMT.

## DISCUSSION

For decades, it has been documented that cancer cells largely rely on glycolysis for their energy metabolism even under aerobic conditions; this is known as the Warburg effect [15]. Because significant glucose uptake and consumption for elevated glycolysis have been observed in many cancer types, these alterations of energy metabolism have been recently considered as an emerging hallmark of cancer [38]. High levels of glycolysis may be beneficial not only to cancer cells by supplying ATP and metabolic byproducts [39], but also for diagnosing cancer using FDG-PET [8, 40]. Recent studies have suggested that FDG uptake reflects increased radioresistance and poor treatment outcomes due to the persistence of cancer cells [11, 12, 41]. Consistently, data from the current study showed that NSCLC cells were more radioresistant in HG media compared to ones maintained in NG media. These observations underscore the importance of glucose not only as an essential nutrient for cancer cells, but also as a reagent which can induce resistance to genotoxic stress such as IR. Although the relevance between glucose levels in tumor tissue niches and the viability of cancer cells has been recognized in recent years, the mechanism and key molecules of glucose-induced radioresistance remain largely unknown.

We found that DANGER expression was specifically induced by HG. It was also observed that DANGER knockdown was effective on just IR response (Figure 2B). We believed that DANGER can help cancer cells to be resistant to radiotherapy similar to oncogenes. However, DANGER is usually expressed at low levels in lung cancer tissues compared with normal lung according to the Oncomine database (www.oncomine.org, Supplementary Figure S8A) [42, 43]. This implies that DANGER itself does not possess oncogenic functions that promote tumorigenesis. We hypothesized that DANGER is indirectly associated with the induction of radioresistance through interplay with signaling pathways of stress-induced cell death. DAPK which promotes apoptosis and functions as a tumor suppressor was found as an interaction partner of DANGER. Data from the Oncomine database showed lower expression of DAPK in lung cancer than normal lung (Supplementary Figure S8B) [44]. We attempted to provide explanations for how DAPK-DANGER interaction and their relevant signaling pathways could increase radioresistance of NSCLC cells in which DAPK is moderately expressed.

We observed that DANGER inhibits the catalytic activity of DAPK through direct binding to the DD of DAPK in NSCLC cells (Figure 4). The DD is a protein-protein interaction-mediating domain that is common to many pro-apoptotic proteins, such as Fas, TNF receptor, TNF receptor type 1-associated death domain protein, and Fas-associated death domain protein. Although no crystal structure of the DD of DAPK has been produced, the DD was previously shown to be involved in interactions between DAPK and several proteins including UNC5H2, TSC2, and kelch-like protein-20 [25]. The DD of DAPK was also found to be necessary to a physical interaction with ERK. ERK phosphorylates DAPK at Ser734, thereby increasing DAPK catalytic activity both *in vitro* and *in vivo* [26]. We found that the pro-apoptotic activity of IR-exposed DAPK, but not the S734A mutant, was markedly reduced by DANGER, and the DD is critical for interaction between DAPK and DANGER (Figure 5). Our results demonstrating enhanced recruitment of DANGER to the ERK-DAPK complex in HG-treated cells compared to NSCLC cells in NG media suggest a specific mode of binding. We propose that ERK and DANGER may compete for binding to the DD of DAPK in HG-treated cells following irradiation. In the HG microenvironment, overexpressed DANGER is able to bind to DAPK and results in decreased ERK-mediated death of IR-irradiated NSCLC cells. Thus, there may be two different modes of complex formation with DAPK based on glucose concentration.

We also found that HG induced anoikis-resistance and EMT activation (Figure 5). Anoikis, a type of programmed cell death caused by detachment of cells from the extracellular matrix, is a defensive mechanism for suppressing anchorage-independent cell growth and EMT involved in cancer metastasis [45, 46]. Although targeting anoikis-resistance is a promising strategy for inhibiting tumor progression, searching for relevant factors and identifying their mechanistic implications would be important for counteracting anoikis-resistance and preventing metastasis. Our data not only provide new evidence that DAPK induces IR-activated anoikis in NSCLC cells, but also demonstrate the presence of an HG-induced anoikis-resistance signaling through which DAPK is inhibited by DANGER. Suggested roles of DAPK and DANGER on potentially influencing metastasis in our results may have significant clinical implications since the EMT and metastasis represent impediments to effective cancer therapy. In addition, we observed that migration of NSCLC cells was significantly induced by irradiation (Figure 5H and Supplementary Figure S6) with which a high level of anoikis-dependent apoptosis was also observed. It has been suggested that IR increases the expression of EMT-inducing proteins to facilitate primary cancer cell survival [47]. The dose of IR used in our study was possibly lethal and activated apoptotic pathways, including the ERK-DAPK-p53 axis. Thus, irradiation-alone could cause a significant level of cell death and increased migration capacity of NSCLC cells which survived after irradiation. However, HG can inhibit DAPK-induced apoptotic signaling through DANGER-DAPK interaction, leading to EMT acceleration and acquisition of radioresistance in NSCLC cells.

**Figure 7:**
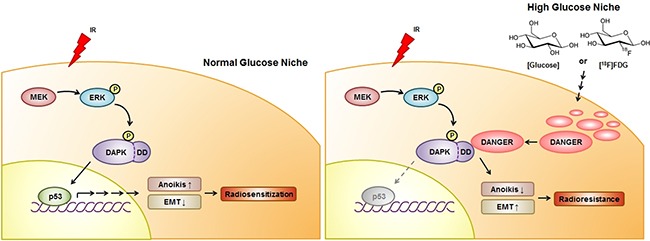
A schematic diagram illustrating MEK-ERK-DAPK-p53 signaling in IR-induced anoikis and the radioresistant effect of DANGER in HG-treated NSCLC cells With NG media, DAPK is phosphorylated on Ser734 by ERK after radiation exposure, consequently leading to the activation of p53 and radiosensitization. IR-induced activation of the MEK-ERK-DAPK-p53 signaling axis is required for anoikis in NSCLC cells. DANGER is overexpressed in NSCLC cells cultured in HG medium, but not in ones maintained under NG conditions. In HG media, overexpressed DANGER directly binds to the DD of DAPK, thus inhibiting the catalytic activity of DAPK. In addition, inhibition of the DAPK-p53 signaling axis by DANGER promotes anoikis-resistance and EMT induction, resulting in radioresistance of HG-treated NSCLC cells. Knockdown of DANGER expression can enhance anoikis, EMT inhibition, and radiosensitization both *in vitro* and *in vivo*.

Although FDG-PET plays a key part in oncological imaging, radioresistance caused by FDG uptake in tumors is a barrier for efficient radiotherapy. Results of the current study showed that DANGER is a HG-associated biomarker and we subsequently performed *in vitro*/*in vivo* analyses to investigate the underlying mechanism of radioresistance induced by DANGER in HG-treated NSCLC. Our results showed that DANGER helps prevent the development of an IR-induced apoptotic phenotype by blocking the MEK-ERK-DAPK-p53 axis (Figure 7). DANGER was found to bind with DAPK, leading to inhibition of DAPK kinase activity, induction of anoikis-resistance, and promotion of EMT *in vitro* and *in vivo*. In addition, we provided the first evidence of a novel regulatory mechanism of radioresistance including the functional involvement of DAPK/ERK or DAPK/DANGER in irradiated NSCLC cells depending on glucose concentration. Taken together, our results demonstrated that suppression of DANGER expression and/or DAPK-DANGER interaction through treatment of specific agents in combination with radiotherapy in FDG-avid tumors could improve the therapeutic efficacy for treating NSCLC by overcoming radioresistance.

## MATERIALS AND METHODS

### Cell lines, cell culture, and irradiation

NCI-H460 and A427 cells were obtained from American Type Culture Collection (Manassas, VA), authenticated, and maintained in early passages, no more than 6 mo after receipt. The cells were grown at 37°C in RPMI-1640 medium containing 10% fetal bovine serum, 100 U/mL penicillin, and 100 μg/mL streptomycin. For glucose treatment, the cells were divided into the following groups: no glucose (NO), NG (5.5 mM), mannitol control (MC, 5.5 mM D-glucose + 24.5 mM mannitol), and HG (30 mM). The cells were then exposed to a single dose of γ-rays using a Gamma Cell-40 Exactor (Nordion International, Inc., Kanata, Ontario, Canada) at a dose rate of 0.81 Gy/min. Flasks containing the control cells were put in the irradiation chamber but not irradiated.

### MTT assay

The effect of DANGER knockdown on cell growth was assessed with an MTT assay as previously described [48]. Cells were cultured in 96-well plates (1,000 cells per well). Cells were transfected with Scrambled or DANGER-specific siRNA for 24 h and then treated with HG for 24 h. After 6 h being exposed to IR, the cells were subjected to an MTT assay. Media were removed and 0.05% MTT solution was added before the cells were incubated at 37°C for 2 h. The MTT solution was subsequently replaced with dimethyl sulfoxide and the plates were incubated for 10 min. After incubation, the solution was aliquoted into 96-well plates in duplicate and absorbance was measured at 570 nm.

### Colony forming assay

Cells were plated at a density of 300 cells in 6-well dishes. After 24 h of glucose treatment, the cells were exposed to a specific dose of radiation, and subsequently grown for 14 d. Next, the cells were fixed with 10% methanol and 10% acetic acid, and stained with 1% crystal violet. Colonies containing more than 50 cells were identified using densitometric software and scored as survivors [49].

### BiFC assay

N-terminal Venus-conjugated EGFR (pBiFC-EGFR-VN) and C-terminal Venus-conjugated EGFR (pBiFC-EGFR-VC) constructs were kindly provided by Dr. Chang-Deng Hu (Purdue University, West Lafayette, IN) and Dr. Ichi N. Maruyama (Okinawa Institute of Science and Technology Graduate University, Okinawa, Japan). To construct pBiFC-DAPK-VN, a DAPK-encoding DNA fragment was amplified by PCR and then inserted into the *Kpn*I site of pBiFC-EGFR-VN, where the region encoding EGFR had been located. To construct pBiFC-DANGER-VC, a DNA fragment encoding DANGER was amplified and inserted between the *Sal*I and *Xho*I sites of pBiFC-EGFR-VC, thus eliminating the EGFR DNA fragment. NCI-H460 or A427 cells were transiently transfected with pBiFC-DAPK-VN and pBiFC-DANGER-VC, and fluorescence was measured with an Olympus IX71 fluorescence microscope (Olympus Optical Co. Ltd., Tokyo, Japan).

### DAPK kinase assay

DAPK kinase activity in cells transfected with FLAG-DAPK and HA-DANGER was assayed as described [31]. DAPK-WT proteins (0.5 mg) immunoprecipitated with anti-FLAG antibody and recombinant GST-MLC at kinase:substrate molar ratios of ~1:5 were incubated with kinase buffer containing 50 mM HEPES (pH 7.5), 8 mM MgCl_2_, 2 mM MnCl_2_, 0.5 mM CaCl_2_, 50 mM ATP, 10 mCi [γ-^32^P]ATP, 0.1 mg/mL bovine serum albumin, and 1 mM bovine CaM (Sigma, St. Louis, MO) at 25°C. For stoichiometric analysis, kinase assays were performed over a time course ranging from 0 to 40 min. Reactions were terminated by boiling in SDS loading buffer and the proteins were separated on 7.5% acrylamide gels that were stained and dried. Levels of incorporated ATP were measured by phosphorimaging relative to a standard curve of known ATP concentrations.

### Luciferase reporter gene assay

A luciferase assay was performed as previously described [50]. Following HG treatment and co-transfection with 3 μg of a p53 luciferase reporter gene (pGL3-luc) or DANGER siRNA, the medium was changed and cells were irradiated. After 3 h, the cells were washed twice with cold PBS and lysed in reporter lysis buffer (Promega, Madison, WI). The lysates were then washed and centrifuged at 12,000 g for 1 min at 4°C. 20 μL of the cell extract and 100 μL of luciferase assay reagent were mixed at room temperature. Samples were placed in Glomax multi-detection system (Promega) to measure luciferase activity in the solution.

### Tumor xenograft in nude mice

For *in vivo* delivery of shRNA into tumors, control or DANGER-specific shRNA were first encapsulated into DOTAP-cholesterol (DC; Avanti Polar Lipids, Inc., Alabaster, AL) nanoparticles by dissolving in sterilized NG or HG media and mixing with the nanosomes [51]. NG- or HG-adapted 2 × 10^6^ NCI-H460 or A427 cells were then injected into the flank of 6-wk-old male BALB/c athymic nude mice and tumors were allowed to develop. Upon identification of a palpable tumor (with a minimum volume of 200 mm^3^), the DC nanoparticle-encapsulated shRNA duplexes were injected into the tumors every 4 d using insulin syringes at a concentration of 20 μg shRNA/100 mm^3^ of tumor volume. To produce sufficient lentiviral particles for transfection of NSCLC cells, the Lenti-vpak packaging kit (Origene) was used according to the manufacturer's instructions. The untreated control groups were injected with NG or HG media as indicated. The animals were also irradiated with 10 Gy once a week for 4 wk. Tumor length (L) and width (l) were measured with a caliper and tumor volumes were calculated using the formula (L × l^2^)/2. At the end of the treatment period, the animals were euthanized and the tumors were used for biochemical studies.

## SUPPLEMENTARY MATERIALS AND METHODS FIGURES



## References

[1] Koh PK, Faivre-Finn C, Blackhall FH, De Ruysscher D (2012). Targeted agents in non-small cell lung cancer (NSCLC): clinical developments and rationale for the combination with thoracic radiotherapy. Cancer Treat Rev.

[2] Bussink J, van der Kogel AJ, Kaanders JH (2008). Activation of the PI3-K/AKT pathway and implications for radioresistance mechanisms in head and neck cancer. Lancet Oncol.

[3] Nishimura Y, Nakagawa K, Takeda K, Tanaka M, Segawa Y, Tsujino K, Negoro S, Fuwa N, Hida T, Kawahara M, Katakami N, Hirokawa K, Yamamoto N (2007). Phase I/II trial of sequential chemoradiotherapy using a novel hypoxic cell radiosensitizer, doranidazole (PR-350), in patients with locally advanced non-small-cell lung Cancer (WJTOG-0002). Int J Radiat Oncol Biol Phys.

[4] Yang HJ, Youn H, Seong KM, Jin YW, Kim J, Youn B (2013). Phosphorylation of ribosomal protein S3 and antiapoptotic TRAF2 protein mediates radioresistance in non-small cell lung cancer cells. J Biol Chem.

[5] Kim E, Youn H, Kwon T, Son B, Kang J, Yang HJ, Seong KM, Kim W, Youn B (2014). PAK1 tyrosine phosphorylation is required to induce epithelial-mesenchymal transition and radioresistance in lung cancer cells. Cancer Res.

[6] Kim W, Youn H, Kwon T, Kang J, Kim E, Son B, Yang HJ, Jung Y, Youn B (2013). PIM1 kinase inhibitors induce radiosensitization in non-small cell lung cancer cells. Pharmacol Res.

[7] Deppen SA, Blume JD, Kensinger CD, Morgan AM, Aldrich MC, Massion PP, Walker RC, McPheeters ML, Putnam JB, Grogan EL (2014). Accuracy of FDG-PET to diagnose lung cancer in areas with infectious lung disease: a meta-analysis. JAMA.

[8] Ben-Haim S, Ell P (2009). 18F-FDG PET and PET/CT in the evaluation of cancer treatment response. J Nucl Med.

[9] Abramyuk A, Tokalov S, Zophel K, Koch A, Szluha Lazanyi K, Gillham C, Herrmann T, Abolmaali N (2009). Is pre-therapeutical FDG-PET/CT capable to detect high risk tumor subvolumes responsible for local failure in non-small cell lung cancer?. Radiother Oncol.

[10] Brown RS, Leung JY, Kison PV, Zasadny KR, Flint A, Wahl RL (1999). Glucose transporters and FDG uptake in untreated primary human non-small cell lung cancer. J Nucl Med.

[11] Ghooshkhanei H, Treglia G, Sabouri G, Davoodi R, Sadeghi R (2014). Risk stratification and prognosis determination using (18)F-FDG PET imaging in endometrial cancer patients: a systematic review and meta-analysis. Gynecol Oncol.

[12] Wang Z, Chen JQ, Liu JL, Qin XG, Huang Y (2013). FDG-PET in diagnosis, staging and prognosis of pancreatic carcinoma: a meta-analysis. World J Gastroenterol.

[13] Feng M, Kong FM, Gross M, Fernando S, Hayman JA, Ten Haken RK (2009). Using fluorodeoxyglucose positron emission tomography to assess tumor volume during radiotherapy for non-small-cell lung cancer and its potential impact on adaptive dose escalation and normal tissue sparing. Int J Radiat Oncol Biol Phys.

[14] Jeong J, Setton JS, Lee NY, Oh JH, Deasy JO (2014). Estimate of the impact of FDG-avidity on the dose required for head and neck radiotherapy local control. Radiother Oncol.

[15] Gatenby RA, Gillies RJ (2004). Why do cancers have high aerobic glycolysis?. Nat Rev Cancer.

[16] Vander Heiden MG, Cantley LC, Thompson CB (2009). Understanding the Warburg effect: the metabolic requirements of cell proliferation. Science.

[17] van Rossum DB, Patterson RL, Cheung KH, Barrow RK, Syrovatkina V, Gessell GS, Burkholder SG, Watkins DN, Foskett JK, Snyder SH (2006). DANGER, a novel regulatory protein of inositol 1,4,5-trisphosphate-receptor activity. J Biol Chem.

[18] Kang BN, Ahmad AS, Saleem S, Patterson RL, Hester L, Dore S, Snyder SH (2010). Death-associated protein kinase-mediated cell death modulated by interaction with DANGER. J Neurosci.

[19] Miao F, Chen Z, Zhang L, Wang J, Gao H, Wu X, Natarajan R (2013). RNA-sequencing analysis of high glucose-treated monocytes reveals novel transcriptome signatures and associated epigenetic profiles. Physiol Genomics.

[20] Bialik S, Kimchi A (2006). The death-associated protein kinases: structure, function, and beyond. Annu Rev Biochem.

[21] Lin Y, Hupp TR, Stevens C (2010). Death-associated protein kinase (DAPK) and signal transduction: additional roles beyond cell death. FEBS J.

[22] Bajbouj K, Poehlmann A, Kuester D, Drewes T, Haase K, Hartig R, Teller A, Kliche S, Walluscheck D, Ivanovska J, Chakilam S, Ulitzsch A, Bommhardt U (2009). Identification of phosphorylated p38 as a novel DAPK-interacting partner during TNFalpha-induced apoptosis in colorectal tumor cells. Am J Pathol.

[23] Chakilam S, Gandesiri M, Rau TT, Agaimy A, Vijayalakshmi M, Ivanovska J, Wirtz RM, Schulze-Luehrmann J, Benderska N, Wittkopf N, Chellappan A, Ruemmele P, Vieth M (2013). Death-associated protein kinase controls STAT3 activity in intestinal epithelial cells. Am J Pathol.

[24] Ivanovska J, Tregubova A, Mahadevan V, Chakilam S, Gandesiri M, Benderska N, Ettle B, Hartmann A, Soder S, Ziesche E, Fischer T, Lautscham L, Fabry B (2013). Identification of DAPK as a scaffold protein for the LIMK/cofilin complex in TNF-induced apoptosis. Int J Biochem Cell Biol.

[25] Bialik S, Kimchi A (2014). The DAP-kinase interactome. Apoptosis.

[26] Chen CH, Wang WJ, Kuo JC, Tsai HC, Lin JR, Chang ZF, Chen RH (2005). Bidirectional signals transduced by DAPK-ERK interaction promote the apoptotic effect of DAPK. EMBO J.

[27] Nakadate Y, Kodera Y, Kitamura Y, Tachibana T, Tamura T, Koizumi F (2013). Silencing of poly(ADP-ribose) glycohydrolase sensitizes lung cancer cells to radiation through the abrogation of DNA damage checkpoint. Biochem Biophys Res Commun.

[28] Li R, Uttarwar L, Gao B, Charbonneau M, Shi Y, Chan JS, Dubois CM, Krepinsky JC (2015). High Glucose Up-regulates ADAM17 through HIF-1alpha in Mesangial Cells. J Biol Chem.

[29] Kim JJ, Tan Y, Xiao L, Sun YL, Qu X (2013). Green tea polyphenol epigallocatechin-3-gallate enhance glycogen synthesis and inhibit lipogenesis in hepatocytes. Biomed Res Int.

[30] Dephoure N, Zhou C, Villen J, Beausoleil SA, Bakalarski CE, Elledge SJ, Gygi SP (2008). A quantitative atlas of mitotic phosphorylation. Proc Natl Acad Sci USA.

[31] Kuo JC, Lin JR, Staddon JM, Hosoya H, Chen RH (2003). Uncoordinated regulation of stress fibers and focal adhesions by DAP kinase. J Cell Sci.

[32] Shohat G, Spivak-Kroizman T, Cohen O, Bialik S, Shani G, Berrisi H, Eisenstein M, Kimchi A (2001). The pro-apoptotic function of death-associated protein kinase is controlled by a unique inhibitory autophosphorylation-based mechanism. J Biol Chem.

[33] Anjum R, Roux PP, Ballif BA, Gygi SP, Blenis J (2005). The tumor suppressor DAP kinase is a target of RSK-mediated survival signaling. Curr Biol.

[34] Chang CC, Yang MH, Lin BR, Chen ST, Pan SH, Hsiao M, Lai TC, Lin SK, Jeng YM, Chu CY, Chen RH, Yang PC, Chin YE (2013). CCN2 inhibits lung cancer metastasis through promoting DAPK-dependent anoikis and inducing EGFR degradation. Cell Death Differ.

[35] Wang WJ, Kuo JC, Yao CC, Chen RH (2002). DAP-kinase induces apoptosis by suppressing integrin activity and disrupting matrix survival signals. J Cell Biol.

[36] Guadamillas MC, Cerezo A, Del Pozo MA (2011). Overcoming anoikis--pathways to anchorage-independent growth in cancer. J Cell Sci.

[37] Huang RY, Wong MK, Tan TZ, Kuay KT, Ng AH, Chung VY, Chu YS, Matsumura N, Lai HC, Lee YF, Sim WJ, Chai C, Pietschmann E (2013). An EMT spectrum defines an anoikis-resistant and spheroidogenic intermediate mesenchymal state that is sensitive to e-cadherin restoration by a src-kinase inhibitor, saracatinib (AZD0530). Cell Death Dis.

[38] Hanahan D, Weinberg RA (2011). Hallmarks of cancer: the next generation. Cell.

[39] Lunt SY, Vander Heiden MG (2011). Aerobic glycolysis: meeting the metabolic requirements of cell proliferation. Annu Rev Cell Dev Biol.

[40] Janssen MH, Aerts HJ, Ollers MC, Bosmans G, Lee JA, Buijsen J, De Ruysscher D, Lambin P, Lammering G, Dekker AL (2009). Tumor delineation based on time-activity curve differences assessed with dynamic fluorodeoxyglucose positron emission tomography-computed tomography in rectal cancer patients. Int J Radiat Oncol Biol Phys.

[41] Xie P, Li M, Zhao H, Sun X, Fu Z, Yu J (2011). 18F-FDG PET or PET-CT to evaluate prognosis for head and neck cancer: a meta-analysis. J Cancer Res Clin Oncol.

[42] Garber ME, Troyanskaya OG, Schluens K, Petersen S, Thaesler Z, Pacyna-Gengelbach M, van de Rijn M, Rosen GD, Perou CM, Whyte RI, Altman RB, Brown PO, Botstein D (2001). Diversity of gene expression in adenocarcinoma of the lung. Proc Natl Acad Sci U S A.

[43] Hou J, Aerts J, den Hamer B, van Ijcken W, den Bakker M, Riegman P, van der Leest C, van der Spek P, Foekens JA, Hoogsteden HC, Grosveld F, Philipsen S (2010). Gene expression-based classification of non-small cell lung carcinomas and survival prediction. PLoS One.

[44] Bhattacharjee A, Richards WG, Staunton J, Li C, Monti S, Vasa P, Ladd C, Beheshti J, Bueno R, Gillette M, Loda M, Weber G, Mark EJ (2001). Classification of human lung carcinomas by mRNA expression profiling reveals distinct adenocarcinoma subclasses. Proc Natl Acad Sci U S A.

[45] Buchheit CL, Weigel KJ, Schafer ZT (2014). Cancer cell survival during detachment from the ECM: multiple barriers to tumour progression. Nat Rev Cancer.

[46] Slattum GM, Rosenblatt J (2014). Tumour cell invasion: an emerging role for basal epithelial cell extrusion. Nat Rev Cancer.

[47] Feofanova N, Geraldo JM, de Andrade LM (2014). Radiation oncology in vitro: trends to improve radiotherapy through molecular targets. Biomed Res Int.

[48] Yang HJ, Youn H, Seong KM, Yun YJ, Kim W, Kim YH, Lee JY, Kim CS, Jin YW, Youn B (2011). Psoralidin, a dual inhibitor of COX-2 and 5-LOX, regulates ionizing radiation (IR)-induced pulmonary inflammation. Biochem Pharmacol.

[49] Niyazi M, Niyazi I, Belka C (2007). Counting colonies of clonogenic assays by using densitometric software. Radiat Oncol.

[50] Kang J, Kim E, Kim W, Seong KM, Youn H, Kim JW, Kim J, Youn B (2013). Rhamnetin and cirsiliol induce radiosensitization and inhibition of epithelial-mesenchymal transition (EMT) by miR-34a-mediated suppression of Notch-1 expression in non-small cell lung cancer cell lines. J Biol Chem.

[51] Kundu AK, Chandra PK, Hazari S, Pramar YV, Dash S, Mandal TK (2012). Development and optimization of nanosomal formulations for siRNA delivery to the liver. Eur J Pharm Biopharm.

