# Supplementary material for: DANGER is involved in high glucose-induced radioresistance through inhibiting DAPK-mediated anoikis in non-small cell lung cancer
Source: Oncotarget. 2016 Jan 12;7(6):7193–206. doi: 10.18632/oncotarget.6887 (PMC4872778; doi:10.18632/oncotarget.6887)
Supplement: Supplementary file 1 [file oncotarget-07-7193-s001.pdf]

SUPPLEMENTARY MATERIALS AND METHODS

Chemicals, antibodies, and reagents

MTT, PD98059 (an ERK inhibitor), and GDC-0973 (a MEK inhibitor) were obtained from Sigma (St. Louis, MO) or EMD-Calbiochem (La Jolla, CA). Antibodies specific for DANGER, Tubulin, pSer/Thr, DAPK, HA, FLAG, myosin regulatory light chain (MLC), pMLC, E-cadherin, Vimentin, Fibronectin, and pERK1 were purchased from Santa Cruz Biotechnology (Santa Cruz, CA), Abcam (Cambridge, UK) or Cell Signaling Technology (Beverly, MA) for Western blotting or IP. Cell culture media (RPMI-1640), fetal bovine serum (FBS), glutamine, penicillin, streptomycin, and Trizol® were obtained from Sigma or Gibco (Grand Island, NY). Control siRNA/shRNA and DANGER-specific siRNA/shRNAs were purchased from Dharmacon (Lafayette, CO) and Santa Cruz Biotechnology.

Real-time quantitative RT-PCR (qRT-PCR)

ITPRIP gene expression was measured using real-time qRT-PCR as previously described [1]. Aliquots of a master mix containing all of the reaction components with the primers were dispensed into a real-time PCR plate (Applied Biosystems, Foster City, CA). All PCR reagents were from a SYBR Green core reagent kit (Applied Biosystems). Sequences of the *ITPRIP*-specific primers were as follows: 5′-AGAACCTGCTGTGTGCCACAGA-3′ (forward), 5′-CAGGTCGAACTCGTACTTGTGG-3′ (reverse). *ITPRIP* gene expression was measured in triplicate in the reaction plate. qRT-PCR was performed using an Applied Biosystems-7900 HT qRT-PCR instrument. PCR was performed for 40 cycles of 95°C for 15 s and 60°C for 1 min followed by thermal denaturation. The expression of each gene relative to that of *GAPDH* was determined using the $2^{-\Delta CT}$ method [2]. To simplify data presentation, relative expression values were multiplied by 10^2.

Western blot analysis, immunoprecipitation (IP), transient transfection, and kinase assay

For Western blot analysis, whole cell lysates (WCL) were prepared as previously described [3]. WCL were extracted using radioimmunoprecipitation assay lysis buffer (50 mM Tris, pH 7.4; 150 mM NaCl, 1% Triton X-100, 25 mM NaF, 1 mM DTT, 20 mM EGTA, 1 mM Na_3VO_4, 0.3 mM PMSF, and 5 U/mL Aprotinin). Protein concentration of the lysates was verified using a Bio-Rad protein assay kit (Bio-Rad Laboratories, Hercules, CA). To prepare the cytoplasmic extract (CE), cells were suspended in buffer A (10 mM HEPES, pH 7.9; 50 mM NaCl, 1 mM DTT, 0.1 mM EDTA, 1 mM PMSF, 1 μg/mL Aprotinin, 5 μg/mL Leupeptin, and 1 μg/mL Pepstatin A) and then incubated for 20 min on ice. An equal volume of buffer B (buffer A + 0.1% NP-40) was then added and the cells were incubated for 20 min on ice. Next, the samples were centrifuged at 5,000 *g* for 2 min to remove cellular debris and the CE was collected. Proteins in the samples were then separated by SDS-PAGE, transferred to a nitrocellulose membrane, and blocked with 5% skim milk in TBS with Tween 20 (10 mM Tris, 100 mM NaCl, and 0.1% Tween 20) for 30 min at room temperature. The membranes were subsequently probed using specific primary antibodies and peroxidase-conjugated secondary antibody (Santa Cruz Biotechnology). Antibody binding was visualized using an enhanced chemiluminescence detection system (Roche Applied Science, Penzberg, Germany).

IP studies were performed as previously described [4]. Briefly, protein samples were immunoprecipitated overnight with specific primary antibody and protein A/G-agarose beads (Santa Cruz Biotechnology). After washing three times with lysis buffer, the immunoprecipitates were boiled in 2 × SDS sample buffer for 10 min followed by centrifugation. The samples were then analyzed by Western blotting.

For transient transfection, cells were plated at a density of 5×10^5 cells in 6-well dishes and incubated for 4 h. Next, the cells were transiently transfected with the indicated plasmid using Lipofectin (Invitrogen, Carlsbad, CA) or siRNA oligonucleotides (10 nM) using DharmaFECT 1 (Dharmacon, Lafayette, CO) according to the manufacturers' instructions.

For the kinase assay, cells were transfected or co-transfected with the indicated gene construct for 24 h. After the desired treatment, the lysates were immunoprecipitated overnight with specific primary antibody. Western blotting was then conducted using phosphor-specific antibody.

Adhesion assay

96-well plates were coated with 10 ng/mL fibrinogen at 4°C overnight, washed with PBS, and blocked with 0.1% BSA for 1 h at 37°C. Cells were suspended in HEPES-Tyrode's buffer containing 0.1% BSA and 1 mmol/L $MgCl_2$, transferred to the plate at a density of 1×10^5 in 200 μL/well, and allowed to attach for up to 60 min at 37°C in 5% CO_2. The plates were washed to remove floating cells. The remaining attached cells were incubated with para-nitrophenol phosphate (5 mg/mL in 50 mmol/L sodium acetate and 1% Triton X-100, pH 5.2) for 30 min and quantified at 405 nm after adding 0.3 mol/L sodium hydroxide.

Apoptosis assay

Apoptosis was assessed by measuring Caspase 3/7 activities using a Caspase-Glo 3/7 assay kit (Promega, Madison, WI) according to the manufacturer's protocol. Briefly, treated cells (10^4 cells/mL) in 100 µL of culture medium were transferred to a 96-well microplate. Subsequently, 100 µL of Caspase-Glo 3/7 reagent containing Caspase 3/7 substrate was added to each well. After contents of the wells were gently mixed at 300–500 rpm for 30 s, the plate was incubated at room temperature for 1 h. Luminescence of each sample was measured using a Glomax multi-detection system (Promega).

Apoptosis induction was also assessed by analyzing cytoplasmic histone-associated DNA fragmentation. In brief, cells were plated in 96-well plates and allowed to attach overnight. The cells were then subjected to transfection, irradiation, and/or drug treatment. Cytoplasmic histone-associated DNA fragmentation was monitored using a cell death detection kit (Roche Applied Science) according to the manufacturer's instructions.

Transwell cell migration assay

To measure the migration capacity of NSCLC cells, a Transwell cell migration assay was conducted as previously described [5]. The assay was performed using a 24-well Transwell chamber (Corning, Inc., Corning, NY). Cells (1×10^4 in serum-free RPMI-1640) cultured in the presence or absence of various conditions (transfection, IR, and/or drugs) for 72 h were seeded in the upper chamber with a 5-µm pore size insert. The lower chamber was filled with 600 µL of RPMI-1640 containing 2% FBS. Six h later, the upper membrane surface was wiped with a cotton swab to remove cells that had not migrated into the lower chamber. Cells that had migrated and attached to the lower membrane surface were fixed with 4% paraformaldehyde and stained with hematoxylin for counting. The migration index was calculated relative to the number of untreated cells that had migrated. The results are expressed as fold-increase of migration compared to the control group and based on the relative number of cells in a randomly selected field for three representative experiments.

Immunofluorescence (IF)

For IF, DAPK- and DANGER-overexpressed cells were grown on glass slides. Following the experimental treatments, the cells were fixed and permeabilized in cold acetone and then washed with cold PBS. After blocking with 1% bovine serum albumin/PBS, the cells were incubated overnight with anti-DAPK and anti-DANGER antibody at 4°C. Next, the cells were washed three times with cold PBS and incubated with DyLight 488-conjugated secondary antibodies and DyLight 649-conjugated ones (Thermo Scientific). After washing and counterstaining with 4′,6-diamidino-2-phenylindole (DAPI) (Sigma), the glass slides were mounted with VECTASHIELD Hard-Set Mounting Medium (Vector Laboratories, Burlingame, CA) and visualized with an Olympus IX71 fluorescence microscope (Olympus Optical Co. Ltd., Tokyo, Japan).

Animal protocol

Six-wk-old male BALB/c athymic nude mice (Central Lab Animals Inc., Seoul, Republic of Korea) were used for the *in vivo* experiments. The protocols used were approved by the Institutional Animal Care and Use Committee of Pusan National University (Busan, Republic of Korea), and performed in accordance with the provisions of the National Institutes of Health Guide for the Care and Use of Laboratory Animals. The mice were housed individually or in groups of up to five in sterile cages. They were maintained in animal care facilities in a temperature-regulated room (23 ± 1°C) with a 12-h light/dark cycle and quarantined for 1 wk prior to the study. The animals were fed water and a standard mouse chow diet *ad libitum*.

Statistical analysis

All numeric data are presented as the mean ± SEM for at least three independent experiments. The results were analyzed using a one-way ANOVA for ranked data followed by a Tukey's honestly significant difference test. Prism 5 software (GraphPad Software, San Diego, CA) was used to perform all statistical analyses. A *p*-value < 0.05 was considered statistically significant.

REFERENCES

1. Kim W, Youn H, Kwon T, Kang J, Kim E, Son B, Yang HJ, Jung Y, Youn B. PIM1 kinase inhibitors induce radiosensitization in non-small cell lung cancer cells. Pharmacol Res. 2013; 70:90–101.
2. Livak KJ, Schmittgen TD. Analysis of relative gene expression data using real-time quantitative PCR and the 2(-Delta Delta C(T)) Method. Methods. 2001; 25:402–408.
3. Yang HJ, Youn H, Seong KM, Yun YJ, Kim W, Kim YH, Lee JY, Kim CS, Jin YW, Youn B. Psoralidin, a dual inhibitor of COX-2 and 5-LOX, regulates ionizing radiation (IR)-induced pulmonary inflammation. Biochem Pharmacol. 2011; 82:524–534.
4. Kim W, Yang HJ, Youn H, Yun YJ, Seong KM, Youn B. Myricetin inhibits Akt survival signaling and induces Bad-mediated apoptosis in a low dose ultraviolet (UV)-B-irradiated HaCaT human immortalized keratinocytes. J Radiat Res. 2010; 51:285–296.
5. Kang J, Kim E, Kim W, Seong KM, Youn H, Kim JW, Kim J, Youn B. Rhamnetin and cirsiliol induce radiosensitization and inhibition of epithelial-mesenchymal transition (EMT) by miR-34a-mediated suppression of Notch-1 expression in non-small cell lung cancer cell lines. J Biol Chem. 2013; 288:27343–27357.

SUPPLEMENTARY FIGURES

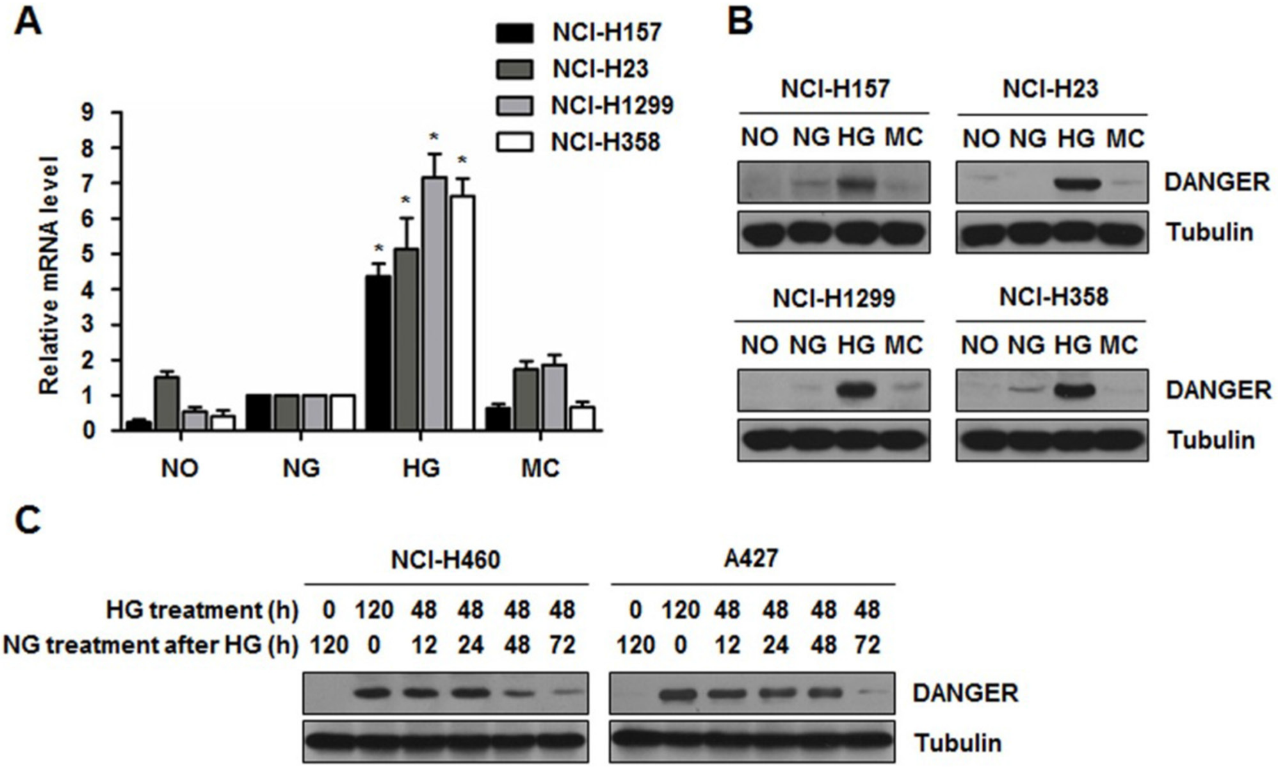

Supplementary Figure S1: HG-induced DANGER overexpression is confirmed in NSCLC cell lines. A. Expression of *ITPRIP* in HG-treated NCI-H157, NCI-H23, NCI-H1299, and NCI-H358 cells was analyzed by qRT-PCR. After treatment with specific media (NO, no glucose; NG, normal glucose; HG, high glucose; or MC, mannitol control) for 24 h, relative mRNA levels of DANGER were monitored. Data are represented as mean ± SEM ($n = 3$); $^{*}p < 0.05$ compared to the NG-treated cells. **B**. Expression of DANGER and time-dependent changes in HG-treated NCI-H157, NCI-H23, NCI-H1299, and NCI-H358 cells were analyzed by Western blotting. **C**. The maintenance of DANGER expression under NG condition after HG treatment in NCI-H460 and A427 cells was assessed by Western blotting.

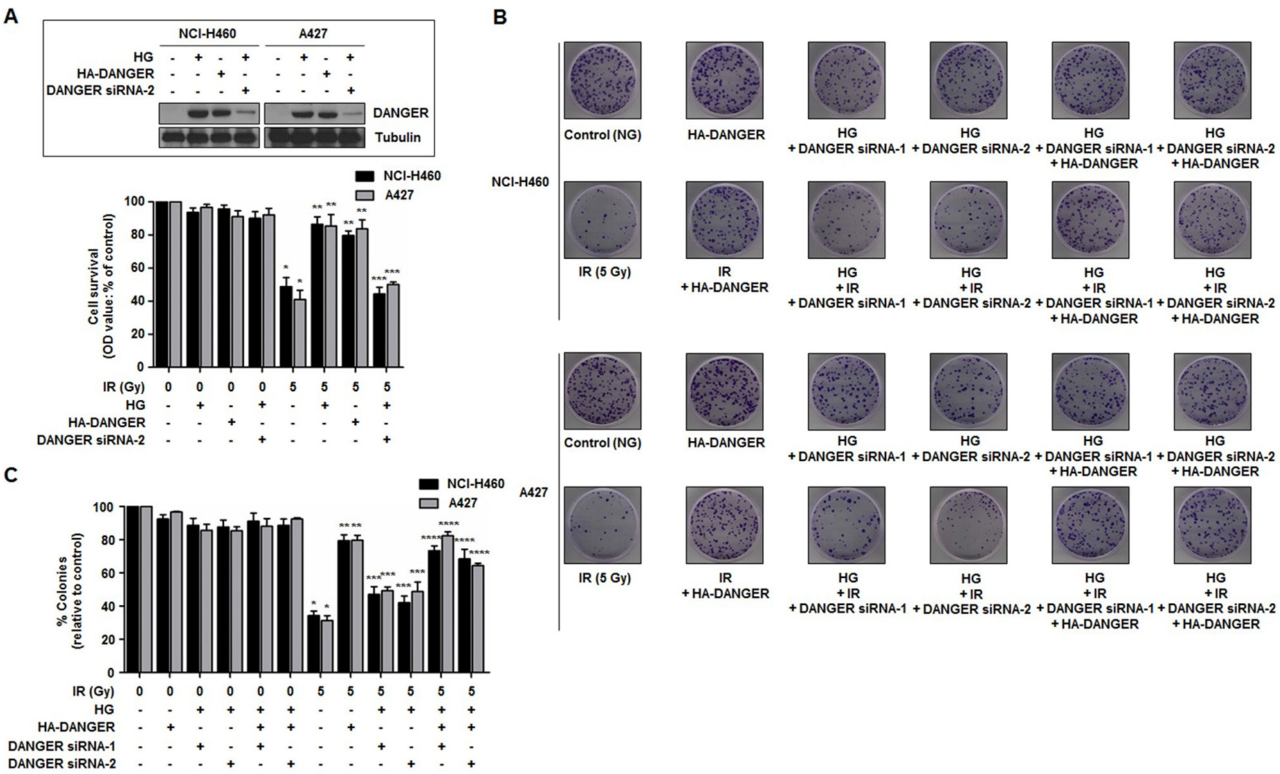

Supplementary Figure S2: Depletion of DANGER decreases radioresistance by HG treatment in NSCLC cells. A. Short-term effects of DANGER knockdown by using DANGER siRNA-2 on cell growth in NCI-H460 and A427 cells following IR exposure were assessed with an MTT assay. Overexpression of DANGER showed similar effect to HG treatment on cell survival of both cell lines after irradiation. Data are represented as mean ± SEM (n = 3); $^{*}p < 0.05$ compared to non-irradiated cells, $^{**}p < 0.05$ compared to cells treated with irradiation-alone, $^{***}p < 0.05$ compared to HG-treated and irradiated cells. Inset: SiRNA knockdown efficiency of DANGER in HG-treated NCI-H460 and A427 cells was analyzed by Western blotting. **B**. Long-term cell growth was confirmed by a colony forming assay. DANGER siRNAs reduced growth of irradiated NCI-H460 and A427 cells treated with HG and overexpression of DANGER reversed the effect. **C**. Quantitative analysis of the number of NCI-H460 and A427 cell clones was performed with Image J. Data are represented as mean ± SEM (n = 3); $^{*}p < 0.05$ compared to non-irradiated cells, $^{**}p < 0.05$ compared to cells treated with irradiation-alone, $^{***}p < 0.05$ compared to DANGER-overexpressed and irradiated cells, $^{****}p < 0.05$ compared to cells treated with HG and DANGER siRNAs.

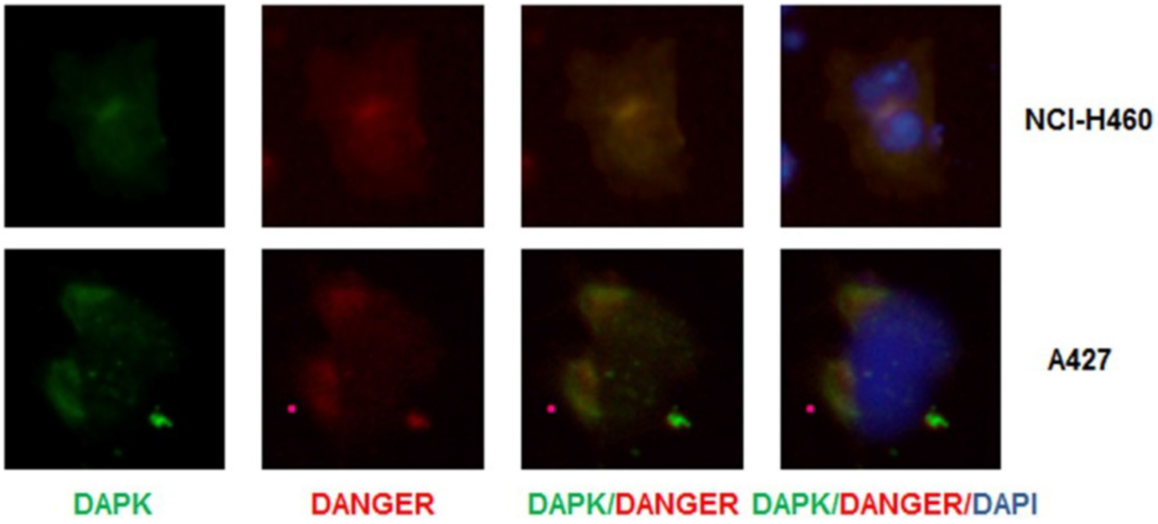

Supplementary Figure S3: Overexpressed DAPK and DANGER co-localize in NCI-H460 and A427 cells. Co-localization of DAPK and DANGER was confirmed by an IF assay. DAPK, DANGER, and cell nuclei are shown as green, red, and blue (DAPI) signals, respectively.

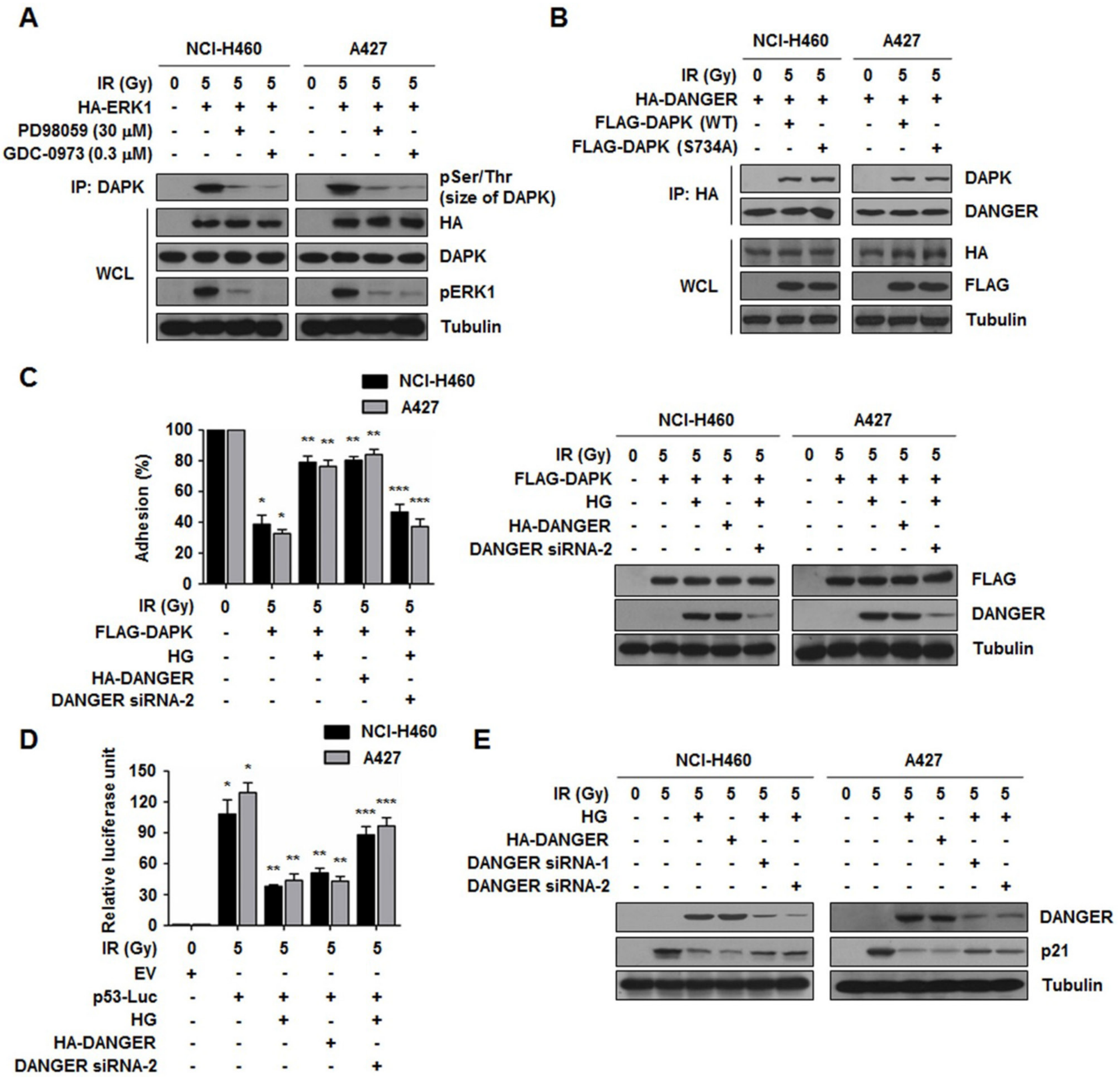

Supplementary Figure S4: ERK1 phosphorylates DAPK and HG treatment or DANGER overexpression increases adhesion and decreases p53 activation in irradiated NSCLC cells. A. DAPK phosphorylation by ERK was confirmed using an ERK inhibitor (PD98059) and a MEK inhibitor (GDC-0973). pERK1 indicates phosphorylated ERK1. **B**. Effect of IR-induced phosphorylation of DAPK on its interaction with DANGER was measured with an IP assay. **C**. Effects of DANGER overexpression on the anti-adhesion activity of DAPK were measured with an adhesion analysis. The cells were irradiated and cell adhesion on fibrinogen was measured. The Western blot results suggested that each transfection was efficient. Data are represented as mean ± SEM ($n = 3$); $^{*}p < 0.05$ compared to non-irradiated cells, $^{**}p < 0.05$ compared to irradiated cells transfected with DAPK, $^{***}p < 0.05$ compared to HG-treated and irradiated cells transfected with DAPK. **D**. Effects of DANGER knockdown on p53 transcriptional activation in HG-treated or DANGER-overexpressed NSCLC cells were measured with a luciferase assay. Data are represented as mean ± SEM ($n = 3$); $^{*}p < 0.05$ compared to non-irradiated cells, $^{**}p < 0.05$ compared to cells treated with irradiation-alone, $^{***}p < 0.05$ compared to HG-treated and irradiated cells. **E**. The expression levels of p21 which is a known target of p53 correlate with the results of p53 luciferase assay in Supplementary Figure S4D.

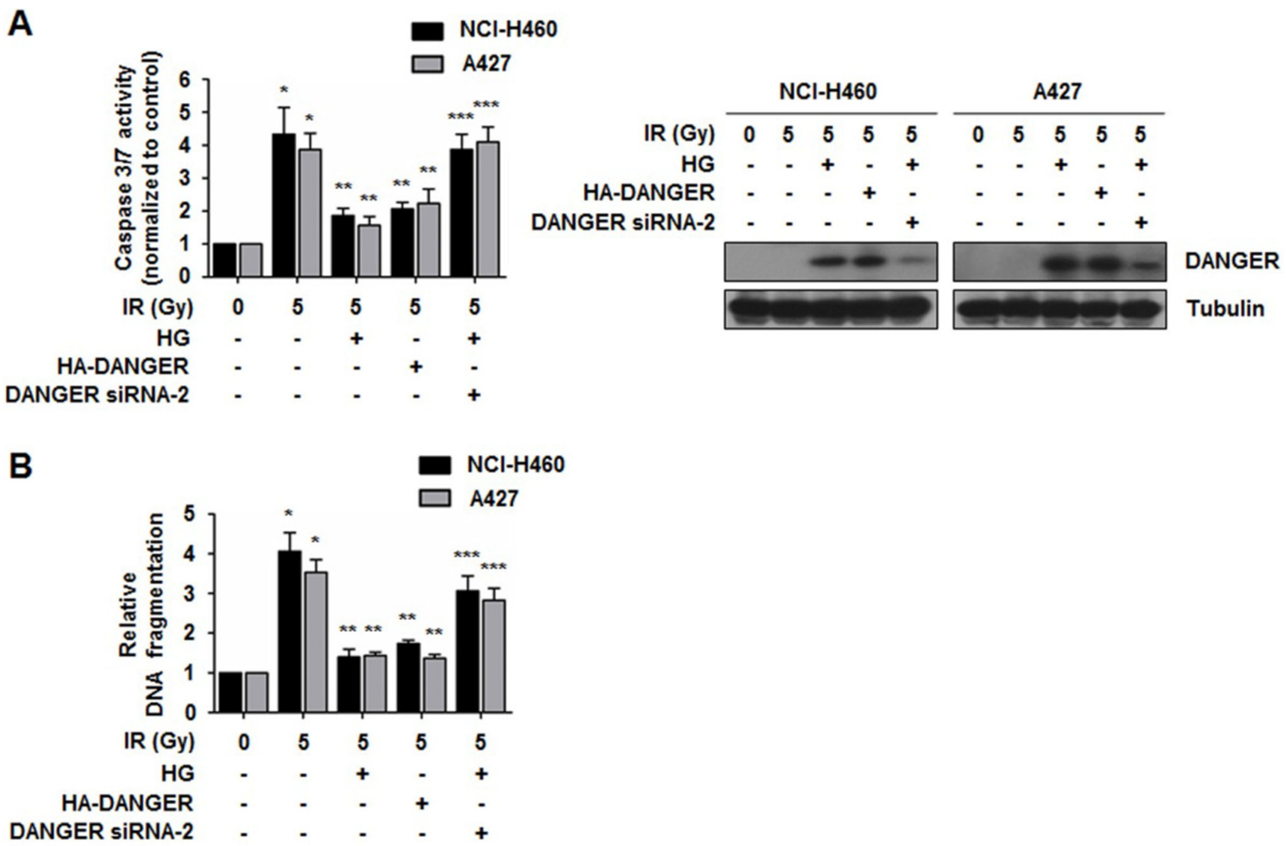

Supplementary Figure S5: The radiosensitivity of NSCLC cells is reduced by HG treatment or DANGER overexpression. A. Caspase 3/7 activation by IR in NSCLC cells were measured with a Caspase 3/7 activity assay. HG- or overexpressed DANGER-induced increase of Caspase 3/7 activity was reversed by DANGER knockdown. The Western blot results suggested that each transfection was efficient. Data are represented as mean ± SEM ($n = 3$); $^{*}p < 0.05$ compared to non-irradiated cells, $^{**}p < 0.05$ compared to cells treated with irradiation-alone, $^{***}p < 0.05$ compared to HG-treated and irradiated cells. **B.** Functional involvement of DANGER knockdown in HG- and IR-induced DNA damage responses was measured with a DNA fragmentation assay. Data are represented as mean ± SEM ($n = 3$); $^{*}p < 0.05$ compared to non-irradiated cells, $^{**}p < 0.05$ compared to cells treated with irradiation alone, $^{***}p < 0.05$ compared to HG-treated and irradiated cells.

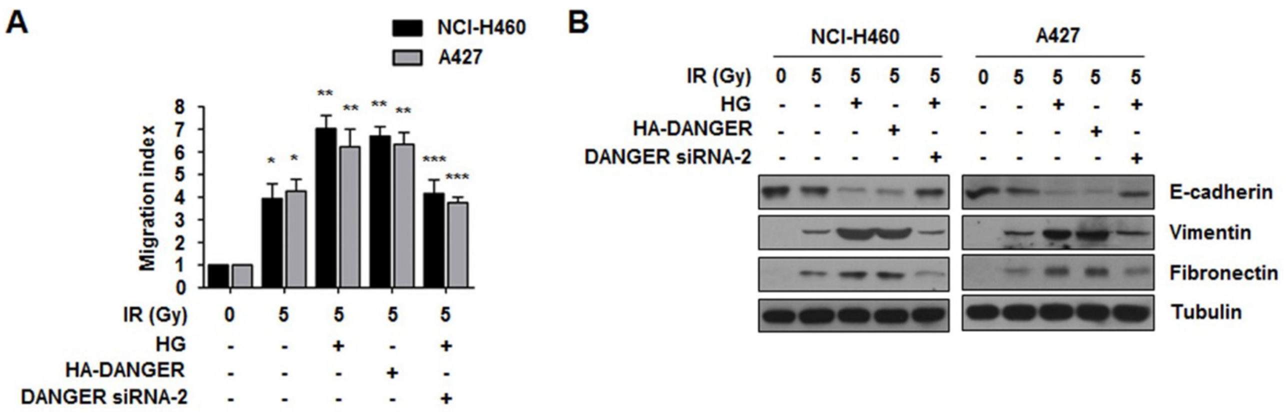

Supplementary Figure S6: DANGER knockdown inhibited EMT increase by HG treatment or DANGER overexpression in irradiated NCI-H460 and A427 cells. **A**. The inhibitory effects of DANGER knockdown on HG- and IR-induced NSCLC cell migration were measured using a Transwell migration assay. Data are represented as mean ± SEM ($n = 3$); $^{*}p < 0.05$ compared to non-irradiated cells, $^{**}p < 0.05$ compared to cells treated with irradiation-alone, $^{***}p < 0.05$ compared to HG-treated and irradiated cells. **B.** Effects of DANGER knockdown on the protein expression of E-cadherin, Vimentin, and Fibronectin in HG-treated and DANGER-overexpressed NSCLC cells were analyzed by Western blotting.

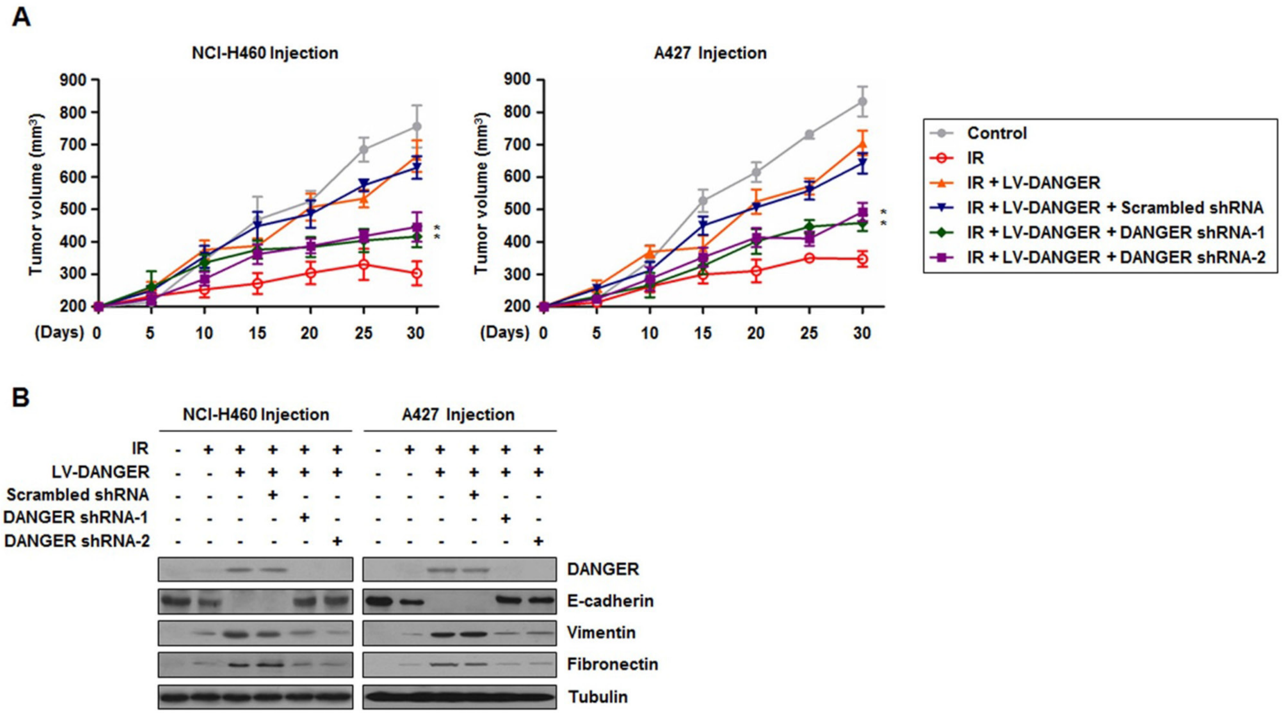

Supplementary Figure S7: Lentiviral overexpression of DANGER increases *in vivo* radioresistance and EMT in a xenograft mouse model. **A.** The effects of DANGER knockdown on *in vivo* radiosensitization were measured in a xenograft mouse model. Data are represented as mean ± SEM ($n = 3$ with three animals/group); $^{*}p < 0.05$ compared to tumor volume on day-30 in mice treated with radiation, lentiviral overexpression of DANGER (LV-DANGER), and Scrambled shRNA. **B.** The *in vivo* effects of DANGER knockdown on the expression of DANGER and EMT-related proteins were evaluated by Western blot analysis.

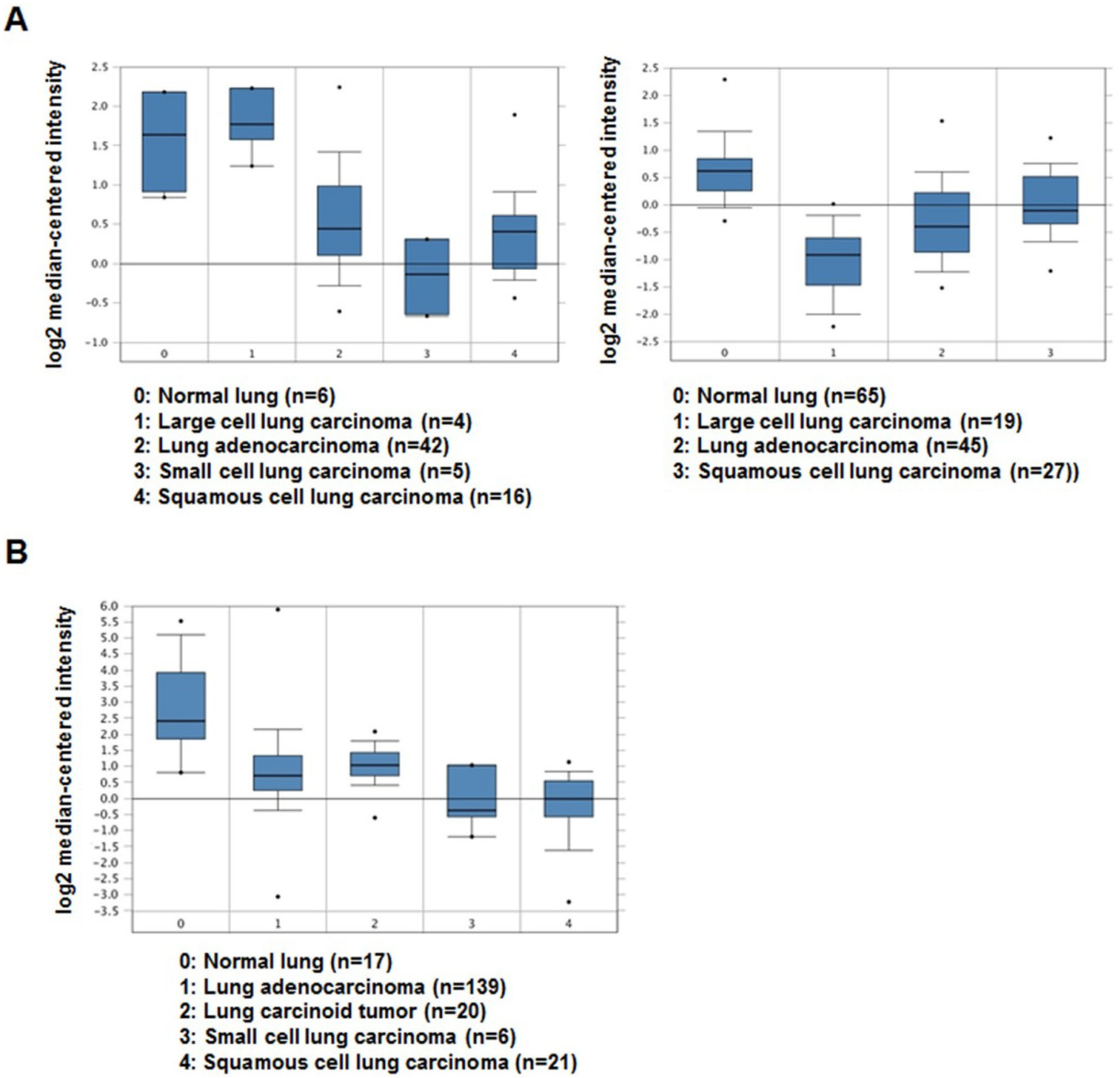

Supplementary Figure S8: Data from the Oncomine database suggests lower expression of DANGER and DAPK in lung cancer compared to normal lung. A. and **B.** Expression of DANGER (A) and DAPK (B) in normal lung and lung cancer tissues was evaluated with data from the Oncomine database (http://www.oncomine.org).
